# The factors associated with subjective cognitive complaints in schizophrenia

**DOI:** 10.1192/j.eurpsy.2021.1453

**Published:** 2021-08-13

**Authors:** M. Bouhamed, R. Sallemi, S. Kolsi, I. Feki, J. Masmoudi

**Affiliations:** 1 Psychiatry, Hedi chaker hospital, Sfax, Tunisia; 2 Psychiatrie “a” Department, Hedi Chaker Hospital University -Sfax - Tunisia, sfax, Tunisia

**Keywords:** cognitif, subjectif, complains, schizophrénia

## Abstract

**Introduction:**

Schizophrenia (SKZ) is a chronic, disabling and incapacitating psychiatric disorder. In addition to the traditional symptomes of schizophrenia, the suffering of this patients can be expressed through a set of cognitive complaints

**Objectives:**

To determe the factors associated with subjective cognitive complaints in schizophrenia

**Methods:**

We conducted a cross-sectional descriptive and analytical study among a sample of of 72 patients followed in psychiatric outpatient of Hefi chaker university hospital in sfax. We used the SSTIC scale to determine subjectif complains ans the PANSS to evalue positif and negatif symptomes

**Results:**

The mean age of our popularion was 46.83± 11.6 years. The patients had a low socio-economic level in 70.1%. They were unemployed in 46.9%, consumed alcohol in 23.6% and consumed tobacco in 58,6% of the cases. the total score on the PANSS scale was 46, distributed as follows: 9 for positive symptoms, 17 for negative symptoms and 22 for total psychopathological assessment. They had an average score of 25 on the total SSTICS score Factors significantly correlated with subjective cognitive complaints were: low socio-economic level (p=0.04), lack of occupation (p=0.001), alcoholism (p=0.001), smoking (p=0.01) and presence of negative symptoms (p=0.00).

**Conclusions:**

This study demonstrates that socio-demographic characteristics and the predominance of negative signs may increase the subjectif cognitif complains in schizophrenia. The recognition of these associations by the psychiatrist can have an important implication in the therapeutic management.

